# Androgen receptor degradation by the proteolysis-targeting chimera ARCC-4 outperforms enzalutamide in cellular models of prostate cancer drug resistance

**DOI:** 10.1038/s42003-018-0105-8

**Published:** 2018-08-02

**Authors:** Jemilat Salami, Shanique Alabi, Ryan R. Willard, Nick J. Vitale, Jing Wang, Hanqing Dong, Meizhong Jin, Donald P. McDonnell, Andrew P. Crew, Taavi K. Neklesa, Craig M. Crews

**Affiliations:** 1 Department of Molecular, Cellular and Developmental Biology, Yale University, New Haven, 06511 CT USA; 2 Department of Pharmacology, Yale School of Medicine, New Haven, 06510 CT USA; 3 Arvinas, LLC, 5 Science Park, New Haven, 06511 CT USA; 4 Department of Pharmacology and Cancer Biology, Duke University School of Medicine, Durham, 27710 NC USA; 5 Department of Chemistry, Yale University, New Haven, 06511 CT USA

## Abstract

The androgen receptor is a major driver of prostate cancer and inhibition of its transcriptional activity using competitive antagonists, such as enzalutamide remains a frontline therapy for prostate cancer management. However, the majority of patients eventually develop drug resistance. We propose that targeting the androgen receptor for degradation via Proteolysis Targeting Chimeras (PROTACs) will be a better therapeutic strategy for targeting androgen receptor signaling in prostate cancer cells. Here we perform a head-to-head comparison between a currently approved androgen receptor antagonist enzalutamide, and its PROTAC derivative, ARCC-4, across different cellular models of prostate cancer drug resistance. ARCC-4 is a low-nanomolar androgen receptor degrader able to degrade about 95% of cellular androgen receptors. ARCC-4 inhibits prostate tumor cell proliferation, degrades clinically relevant androgen receptor point mutants and unlike enzalutamide, retains antiproliferative effect in a high androgen environment. Thus, ARCC-4 exemplifies how protein degradation can address the drug resistance hurdles of enzalutamide.

## Abstract (web summary)

Jemilat Salami et al. develop a proteolysis targeting chimera ARCC-4, which inhibits prostate tumor cell proliferation via degradation of the androgen receptor. They show in cells that ARCC-4 is more effective than the prostate cancer drug enzalutamide and can degrade androgen receptor variants resistant to enzalutamide.

## Introduction

Androgen receptor (AR) signaling is crucial for normal prostate development, but also drives the growth and survival of prostate cancer cells. As such, AR signaling suppression is a common strategy for treating prostate cancer^1^. For example, androgen deprivation therapy (ADT), via either surgical or chemical castration, has been the standard of care for decades^2,3^. Nonetheless, a castration-resistant form of the disease eventually develops, whereby tumor cell proliferation resumes despite sub-castration levels of serum testosterone^2^. More recently, the addition of anti-androgen therapy to ADT, either in the form of AR inhibitors (e.g., enzalutamide)^4^ or CYP17 inhibitors, has improved overall survival^5,6^, although resistance ultimately still develops. As tumors progress, the majority of patients demonstrate AR gene amplifications or mutations, and these two events appear mutually exclusive^7^. Almost all patients also demonstrate a rise in serum prostate specific antigen (PSA) levels^8^, suggesting that AR remains the principal driver of metastatic disease.

Like other occupancy-based inhibitors, the antiandrogen enzalutamide requires high saturating drug concentrations to achieve its clinical benefit^9,10^. Unfortunately, this need to achieve and maintain high systemic concentrations is one of the major challenges in drug development today. However, in recent years, an alternative potential therapeutic approach, i.e., induced protein degradation^11–13^, has emerged and is based on event-driven (as opposed to occupancy-driven) pharmacology. This therapeutic approach is best exemplified by Proteolysis Targeting Chimeras (PROTACs), which are heterobifunctional molecules that work by creating a trimeric complex between a target protein and an E3 ubiquitin ligase, thus facilitating target ubiquitination and subsequent degradation^14–16^. The fact that PROTAC engagement leads to target protein degradation offers a potential advantage over occupancy-based inhibitors.

To explore the potential advantages of a degrader versus an inhibitor, we synthesized a variety of enzalutamide-based von Hippel-Lindau (VHL)-recruiting AR PROTACs and selected our most potent compound ARCC-4 to compare with its parent inhibitor enzalutamide. We demonstrate herein that compared to its parent inhibitor, ARCC-4 is better at overcoming resistance in cellular models of castration-resistant prostate cancer (CRPC). Specifically, ARCC-4 is better than enzalutamide at inducing apoptosis and inhibiting proliferation of AR-amplified prostate cancer cells. Furthermore, ARCC-4 effectively degrades clinically relevant AR mutants associated with antiandrogen therapy, and maintains its potency to degrade AR and inhibit cell proliferation in a high androgen environment in which enzalutamide has no activity. We expect that PROTAC-mediated AR degradation can potentially address a number of AR-dependent mechanisms of drug resistance that are characteristic of castration-resistant prostate cancer but currently not addressed by enzalutamide-mediated inhibition.

## Results

### AR PROTAC potently degrades AR in multiple cancer cell lines

**Fig. 1:**
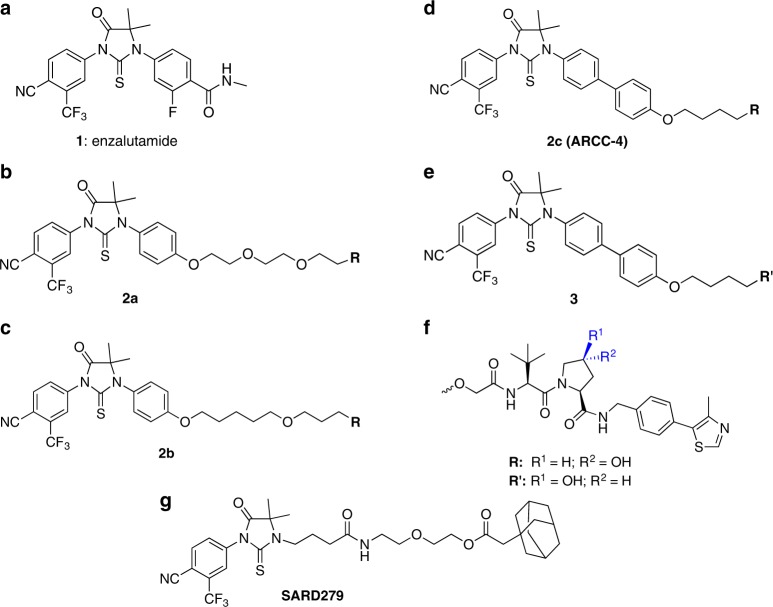
Structures of AR-targeting PROTACs. **a** Chemical structures of enzalutamide. **b**–**d** Chemical structures of AR PROTACs 2a, 2b, and 2c. **e** Chemical structure of inactive epimer, which does not bind VHL. **f** Chemical structure of VHL component of AR PROTACs and epimer (R and R’, respectively), indicating the corresponding stereochemistry. **g** Chemical structure of selective androgen receptor degrader compound, SARD279

We generated AR-targeting PROTACs (compounds 2a–c) by appending enzalutamide to our previously reported VHL E3 ligase ligand^17^ via different linkers (Supplementary Figure 1, Fig. 1). To control for the notable size and cell permeability differences between the PROTACs and enzalutamide, we also designed an epimeric PROTAC (compound 3) by appending enzalutamide to a VHL diastereomeric ligand that does not bind to or recruit VHL for target ubiquitination (Supplementary Figure 1), thereby creating a physicochemically-matched PROTAC analog capable of inhibiting AR but not inducing its degradation. We chose the VCaP cell line to assess the efficacy of our AR PROTACs since it exhibits wild-type AR amplification, splice variant AR-V7 expression, and TMPRSS2-Erg translocation and is a well-validated model of CRPC^18–20^. Although this cell line expresses markers that are predictive of enzalutamide resistance, they do in fact respond to the antiandrogenic actions of this drug at high concentrations^21^.

**Fig. 2:**
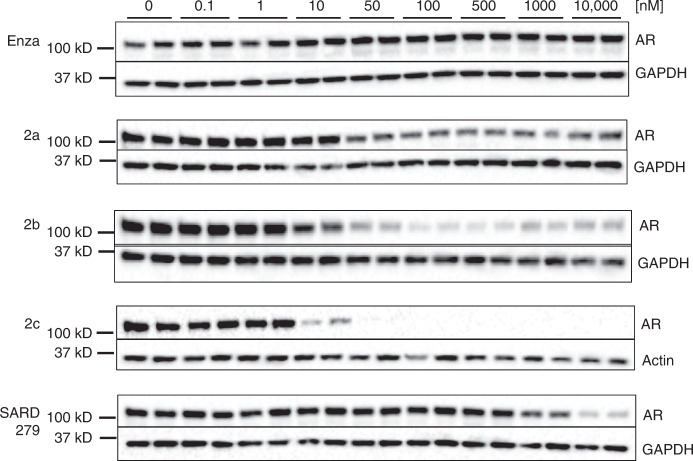
Activity of AR-targeting PROTACs. VCaP cells cultured in charcoal-stripped serum (CSS) were treated with enzalutamide (enza), AR PROTACs (2a, 2b, and 2c) and SARD279 for 20 h. AR levels were determined by western blots. The western blot shows biological replicates and is representative of two independent experiments (*n* = 2). See Supplementary Figure 7 for full blot images

We first treated VCaP cells with increasing concentrations of different AR PROTACs (2a–c) for 20 h and assessed AR levels post-treatment (Fig. 2). We also tested our previously reported selective AR degrader SARD279^22^ in these experiments for comparison. All three AR PROTACs degraded AR, however to different extents; 2a was the least potent, 2b showed better potency, and 2c was the most potent, achieving 50% AR degradation (DC_50_) at 5 nM and a maximum degradation (*D*_max_) of over 95% (Table 1). We also observed the “hook effect” with 2b, in which loss of target degradation is seen at higher concentrations of some PROTACs due to the formation of separate binary complexes of PROTAC-E3 ligase and PROTAC-target protein instead of the productive ternary complex of E3 ligase-PROTAC-target protein^23,24^. We were not surprised at the lack of a hook effect with compound 2c as it is not our first potent PROTAC to not display a hook effect^25^. We suspect that the lack of hook effect with some of our potent PROTACs may be as a result of positive cooperativity, i.e., additional protein–protein interactions between the E3 ligase and target protein, upon ternary complex formation. We hypothesize that ARCC-4 might enhance protein–protein interactions between AR and VHL, thereby promoting the association of the trimeric complex. Given that 2c was the most potent PROTAC, we proceeded with this compound (subsequently denoted as ARCC-4) for further experiments.

**Table 1:** Degradation activity of AR-targeting PROTACs

	*D*_max_	DC_50_ [nM]
Enza	-	-
2a	47%	-
2b	76%	15
2c	98%	5
SARD279	69%	1099

DC_50_ is the concentration of compound required to achieve 50% degradation. *D*_max_ is the maximum degradation achieved by compound. DC_50_ and *D*_max_ values were calculated using quantified band intensities from western blots shown in Fig. 2

**Fig. 3:**
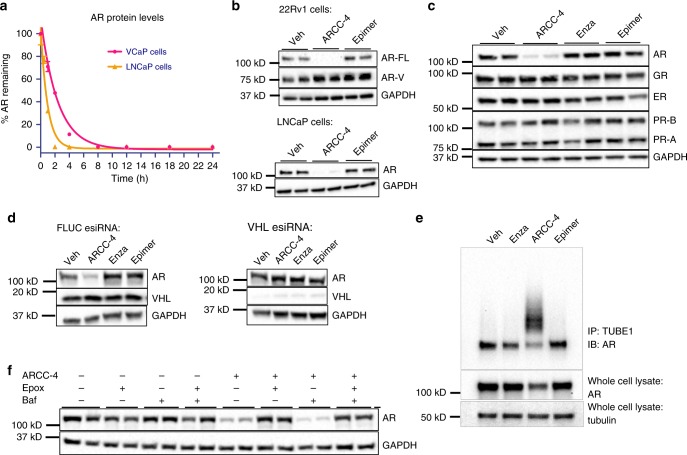
PROTAC ARCC-4 potently degrades AR in prostate cancer cells. **a** Time course serum-free treatment of VCaP and LNCaP cells with 100 nM of ARCC-4 shows over 90% AR depletion by 6 h as measured by quantified western blot. AR levels were normalized to tubulin as a loading control. See Supplementary Figures 2, 3 for western blot images. Experiment was performed in duplicate. The data represent mean values ± SEM and the plot represents two independent experiments (*n* = 2). **b** Treatment of 22Rv1 and LNCaP cells for 24 h with vehicle (Veh), ARCC-4 or epimer (100 nM) in charcoal-stripped serum (CSS) media shows AR depletion as measured by western blot. AR-FL: full length AR; AR-V: splice variants of AR. Experiments were performed in biological duplicates and the plot represents two independent experiments (*n* = 2). See Supplementary Figure 8 for full blot images. **c** Treatment of T47D breast cancer cells for 24 h with 50 nM enzalutamide (Enza) or ARCC-4 shows no loss of glucocorticoid, estrogen, and progesterone receptors. The western blot shows biological replicates and represents two independent experiments (*n* = 2). See Supplementary Figure 8 for full blot images. **d** Transfection of VCaP cells with endo-ribonuclease prepared siRNA (esiRNA) targeting Firefly Luciferase (FLUC) as a negative control, or VHL shows a loss of ARCC-4 mediated AR degradation upon VHL knockdown. VCaP cells were treated with 100 nM of the indicated compounds for 12 h after 48 h post-transfection. Data represents two independent experiments. See Supplementary Figure 9 for full blot images. **e** Tandem Ubiquitin Binding Elements (TUBE1) pull-down assay in VCaP cells shows enrichment of polyubiquitin chains on AR upon a 2.5-hour treatment with 1 µM ARCC-4. This polyubiquitination of AR is not seen with the other compounds tested. Data represents two independent experiments. See Supplementary Figure 9 for full blot images. **f** ARCC-4-mediated AR degradation (1 µM) at 4 h is blocked by 1-h pretreatment with proteasome inhibitor epoxomicin (Epox, 2 µM) and is unaffected by 1-h pretreatment with lysosomal inhibitor bafilomycin (Baf, 100 nM). The western blot shows biological replicates and represents two independent experiments (*n* = 2). See Supplementary Figure 9 for full blot images

Following our observations of AR degradation in VCaP cells after 20 h of PROTAC treatment, we sought to investigate the kinetics of this degradation. VCaP cells were treated with 100 nM ARCC-4 for different lengths of time and AR degradation was assessed by western blotting (Supplementary Figure 2, Fig. 3a). Approximately 90% of AR was degraded in these cells by 4 h, and we observed near complete AR degradation (>98%) within 12 h of treatment. Similar degradation kinetics were observed in LNCaP prostate cancer cells (Supplementary Figure 3, Fig. 3a).

We next sought to identify other cellular contexts aside from VCaP and LNCaP cells in which ARCC-4 could induce AR degradation. We therefore tested another prostate cancer cell line (22Rv1) and a breast cancer cell line (T47D) and found that ARCC-4 efficaciously degrades AR in all of these cell lines (Fig. 3b, c). These results suggest that ARCC-4 could also be utilized in other cancer cell types for which AR degradation might confer a therapeutic benefit.

### ARCC-4 selectively degrades AR via the proteasome

Having established the potency of ARCC-4-induced degradation, we next explored the mechanism of this AR degradation. The lack of degradation with our ARCC-4 epimer (Fig. 3b) and upon VHL knockdown (Fig. 3d) confirmed that ARCC-4-mediated AR degradation is dependent upon VHL recruitment and binding. Furthermore, we performed a Tandem Ubiquitin Binding Element (TUBE1) pull-down assay that shows ARCC-4 induces AR polyubiquitination that subsequently leads to its degradation (Fig. 3e). To determine the specific mechanism of PROTAC-induced AR degradation, we pre-treated VCaP cells with the proteasomal inhibitor epoxomicin^26^, the lysosomal inhibitor bafilomycin^27^ or both (Fig. 3f). Our data show that AR degradation was blocked by epoxomicin, but was unaffected by bafilomycin, suggesting that ARCC-4 induces the degradation of AR in cells via the proteasome.

We next sought to explore the selectivity of ARCC-4 for AR degradation over other nuclear hormone receptors of similar homology—namely the estrogen, progesterone, and glucocorticoid receptors^28^. To assess the selectivity of ARCC-4 for AR, we examined the levels of these receptors in T47D cells that had been treated with the compound. Upon treatment with a high concentration of compound (1 μM), we found that ARCC-4 had no effect on glucocorticoid receptor and estrogen receptor levels, while there was a modest decrease in progesterone receptor (PR) A and B levels in these cells (Supplementary Figure 4). However, at lower concentrations of ARCC-4 that still induce potent AR degradation, we no longer see any effects on PR-A and PR-B levels (Fig. 3c). These data are consistent with the observation that enzalutamide analogs have high affinity for AR, weak affinity for PR and no detectable binding towards the glucocorticoid and estrogen receptors^21,29^. Moreover, the prostate cancer cells used in this study do not express detectable levels of the PR and as such, any ARCC-4 induced signaling or proliferation effects can be solely attributed to AR degradation and not PR-A or PR-B suppression.

### ARCC-4 shows functional gains over enzalutamide

**Fig. 4:**
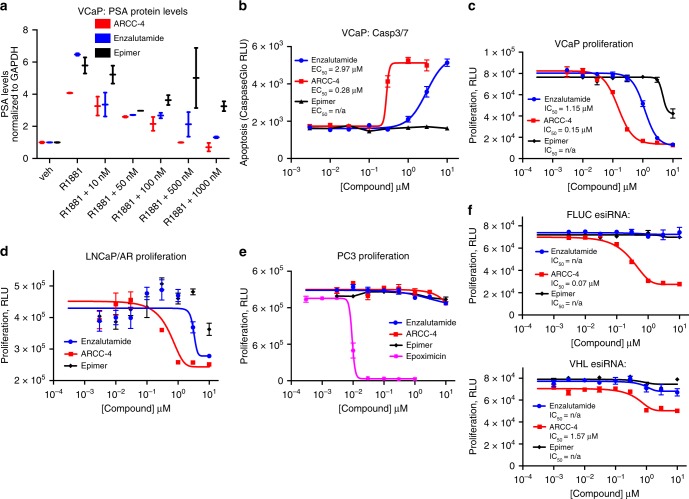
ARCC-4 is better than enzalutamide in inducing apoptosis and inhibiting AR signaling in prostate cancer cells overexpressing AR. **a** Pretreatment of VCaP cells with ARCC-4, enzalutamide or epimer for 8 h in charcoal-stripped serum (CSS) media followed by a 48-h stimulation with 0.2 nM R1881 shows blockage of PSA upregulation as measured by western blot. Experiments were performed in duplicate. The results are representative of two independent experiments (*n* = 2). **b** Treatment of VCaP cells for 48 h with enzalutamide, ARCC-4, or epimer leads to varying apoptosis induction as measured by caspase-3 and caspase-7 activation. Experiments were performed in triplicate and the plot is representative of two independent experiments (*n* = 2). **c**, **d** Treatment of CRPC cells (VCaP or LNCaP/AR) with enzalutamide, ARCC-4, or epimer for 6 days demonstrates antiproliferative effects. Experiments were performed in triplicate and the plot is representative of two independent experiments (*n* = 2). **e** Treatment of AR-negative PC3 prostate cancer cells with enzalutamide, ARCC-4, or epimer for 6 days shows no antiproliferative effect. Epoxomicin treatment was a positive control for toxicity in these cells. Experiments were performed in triplicate and the plot is representative of two independent experiments (*n* = 2). **f** Treatment of VCaP cells with enzalutamide, ARCC-4, or epimer for 6 days in 0.1 nM R1881, following a 48-h FLUC or VHL esiRNA transfection. Data shows a substantial reduction in ARCC-4 inhibition of cell proliferation upon VHL knockdown. Experiments were performed in triplicate. All data represent mean values ± SEM

In line with current considerations regarding the therapeutic benefits of PROTAC-mediated targeted degradation over inhibition^11–13^, we sought to examine these potential advantages by comparing ARCC-4 and enzalutamide in the context of CRPC resistance mechanisms. One such resistance mechanism is AR overexpression in prostate tumor cells, which is replicated in the VCaP and LNCaP/AR cell lines through gene amplification and genetically engineered stable overexpression, respectively. We first tested the effect of ARCC-4 on AR downstream signaling by measuring its ability to block PSA upregulation upon androgen stimulation by the synthetic androgen R1881. We found that an 8-h pretreatment with this PROTAC was sufficient to block androgen induced PSA upregulation in VCaP cells (Fig. 3a). As expected, we observed the same result with enzalutamide but found the epimer to be much less potent at blocking PSA upregulation (Fig. 4a). We next examined the ability of our compounds to induce apoptosis in CRPC cells by measuring levels of caspase-3 and caspase-7, two proteins that are proteolytically activated during the induction of apoptosis^30,31^. ARCC-4 induced apoptosis with an EC_50_ 10-fold lower than that of enzalutamide, while the control epimer PROTAC had no effect on the apoptosis induction (Fig. 4b). Following the differential potencies of ARCC-4 and enzalutamide in inducing CRPC cell apoptosis, we next investigated the consequences of AR inhibition and degradation on the proliferation of AR-amplified CRPC cells. In agreement with our preceding data, ARCC-4 inhibited the growth of VCaP and LNCaP/AR cells at lower concentrations than enzalutamide (Fig. 4c, d). We also observed similar results in LNCaP cells (Supplementary Figure 5). To investigate these observed differences in activity between ARCC-4, enzalutamide, and the epimer, we used an AR radioligand binding assay to determine the IC_50_ values for inhibiting binding between AR and (^3^H) R1881. We found that the IC_50_ values for ARCC-4, enzalutamide, and epimer were comparable at 36 nM, 70 nM, and 41 nM, respectively (Supplementary Table 1). Essentially, we were unable to assign our observed differences in potency to differing AR binding affinities. Furthermore, although the epimer and enzalutamide both engage AR comparably, we believe the epimer’s lower potency may be due to its larger molecular weight that could be an impediment to intracellular accumulation. Along the same line, given that the epimer shares the same physico-chemical properties as ARCC-4, we postulate that the more potent biological activity associated with ARCC-4 is derived from its ability to degrade AR.

To verify that growth inhibition in our previously tested cells was due to the specific disruption of AR signaling, we treated AR-negative PC3 prostate cancer cells with both ARCC-4 and enzalutamide (Fig. 4e). We found that neither enzalutamide nor ARCC-4 affected the growth of these cells, confirming that these compounds are not pan cytotoxic, and that their activity can be directly attributed to their ability to disrupt AR signaling. We also show that the enhanced ability of ARCC-4 to inhibit CRPC cell proliferation is as a result of PROTAC-mediated AR degradation. We transfected VCaP cells with either FLUC or VHL esiRNA for 48 h and then treated these cells with the different compounds. We found that VHL knockdown and the resulting disruption of ARCC-4-induced protein degradation led to a substantial reduction in the inhibition of CRPC cell proliferation by ARCC-4 (Fig. 4f), and the potency difference between ARCC-4 and the other test compounds. Also, we believe the residual inhibitory activity observed with the VHL knockdown cells is probably due to incomplete VHL knockdown given that these data were collected at a time-point that was 1 week after esiRNA transfection. These data suggest that the large potency difference between ARCC-4 and enzalutamide/epimer can be attributed to induced protein degradation.

### ARCC-4 effectively degrades clinically relevant AR mutants

**Fig. 5:**
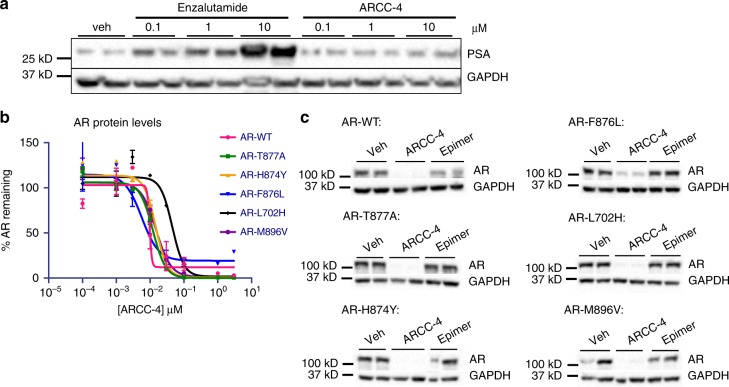
ARCC-4 shows efficacy against clinically relevant AR mutations. **a** Treatment of LNCaP/F876L AR cells with indicated concentrations of enzalutamide or ARCC-4 for 48 h shows a greater increase in PSA levels with enzalutamide as measured by western blot. Results are representative of two independent experiments (*n* = 2). veh refers to vehicle-treated samples. See Supplementary Figure 10 for full blot images. **b** Treatment of HEK293T cells overexpressing wild-type (WT) or different AR mutants for 20 h (after 50 ng per ml doxycycline induction) with ARCC-4 in charcoal-stripped serum (CSS) media shows AR depletion of WT and all mutants tested as measured by quantified western blot. AR levels were normalized to GAPDH as a loading control. See Supplementary Figure 6 for western blot images. Experiments were performed in duplicate and the plot is representative of two independent experiments (*n* = 2). **c** AR degradation in HEK293T cells overexpressing WT or different AR mutants treated for 20 h (after 50 ng per ml doxycycline induction) with vehicle, ARCC-4, or epimer (500 nM) in CSS media. Experiment was performed in biological duplicates (*n* = 1). See Supplementary Figure 10 for full blot images. All data represent mean values ± SEM

Various AR mutations have been observed in clinical samples from metastatic prostate cancer patients. Some of these AR mutations (e.g., F876L and T877A)^32^ alter the activity of known AR antagonists by changing them into agonists, while others allow for promiscuous activation of AR via other hormone ligands (Table 2). Consequently, we set out to determine whether our AR PROTAC could target and degrade AR mutants of pathological interest. Given the ability of the AR-F876L mutant to mediate enzalutamide resistance by causing enzalutamide to function as an agonist, we first determined whether these inhibitor-refractory cancer cells would be vulnerable to protein degradation. LNCaP cells engineered to overexpress the mutant AR-F876L (LNCaP/F876L) were treated with increasing concentrations of enzalutamide or ARCC-4 in the absence of androgens. As expected, the PSA levels in the enzalutamide-treated cells increased substantially (~ 17.5-fold at 10 µM), whereas the increase observed upon treatment with ARCC-4 was reduced (~ 3.5-fold at 10 µM) (Fig. 5a). These results suggest that enzalutamide-based AR PROTACs pose a decreased agonism liability in these mutant cells. Based on the promising results we obtained for the AR-F876L mutation, we examined other AR point mutations (H874Y, M896V, T877A, L702H) observed in patients exposed to AR-targeted therapies^33,34^. Since these AR mutants are thought to retain binding to AR antagonists, we speculated that ARCC-4 could successfully degrade these mutated forms of AR. For this experiment, we used HEK293T cells engineered to stably express WT or the different clinically relevant AR mutants. We performed dose response treatments for each of the cell lines and found that ARCC-4 degraded all of the AR mutants tested (Supplementary Figure 6, Fig. 5b, c). These data suggest that ARCC-4 is able to target clinically relevant mutants of AR; and that protein degradation could have therapeutic benefits in mutational contexts that are not susceptible to AR inhibition.

**Table 2:** List of some drug-resistant AR mutations and their corresponding alternative (steroid hormone or antiandrogen) agonists

AR mutation	Substitute agonist	Reference
F876L	Enzalutamide, ARN 509	Balbas et al. (2013)^46^
T877A	Flutamide, glucocorticoids, progesterone	Fenton et al. (1997)^47^
		Taplin et al. (1999)^48^
		Veldscholte et al. (1990)^49^
H874Y	Flutamide, glucocorticoids, progesterone	Fenton et al. (1997)^47^
		Tan et al. (1997)^50^
M896V	Bicalutamide	Liu et al. (2015)^51^
L702H	Glucocorticoids, cortisol, cortisone	Zhao et al. (2000)^52^
W741L	Bicalutamide	Hara et al. (2003)^53^

### ARCC-4 maintains activity despite elevated androgen levels

**Fig. 6:**
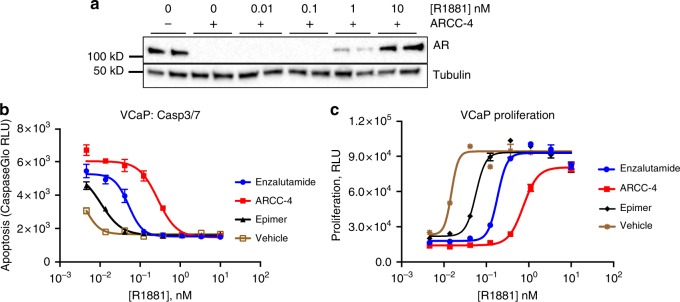
ARCC-4 retains activity and outperforms enzalutamide in the presence of higher androgen levels. **a** Treatment of VCaP cells with ARCC-4 (1 µM) in charcoal-stripped serum (CSS) media for 6 h shows reversal of AR degradation with increasing amounts of synthetic androgen R1881. This experiment is representative of two independent experiments (*n* = 2). See Supplementary Figure 11 for full blot images. **b** AR PROTAC promotes apoptosis at concentrations of R1881 that overcome enzalutamide-induced apoptosis. VCaP cells were treated for 48 h with increasing amounts of R1881 in the presence of vehicle, enzalutamide, ARCC-4, or epimer (all 1 µM); apoptosis induction was measured by activation of caspase 3/7. Experiments were performed in triplicate and the plot is representative of two independent experiments (*n* = 2). **c** AR PROTAC blocks prostate cancer cell proliferation at concentrations of R1881 that overcome enzalutamide-induced growth arrest. VCaP cells were treated for 6 days with increasing amounts of R1881 in the presence of vehicle, enzalutamide, ARCC-4, or epimer (all 1 µM). Experiments were performed in triplicate and the plot is representative of two independent experiments (*n* = 2). All data represent mean values + SEM

It is thought that some prostate tumors adapt to low serum androgen levels by upregulating genes responsible for making intratumoral androgens via paracrine and autocrine mechanisms, thus bypassing the need for systemic androgens^35,36^. Because enzalutamide competes with androgens for binding to AR ligand binding domain, elevated androgen levels lead to enzalutamide resistance^21^. Even though ARCC-4 shares the enzalutamide moiety, we speculated that an AR PROTAC can induce AR degradation since PROTACs only require momentary target interaction to tag a protein for degradation. We were thus interested in comparing the efficacy of ARCC-4 and enzalutamide in inhibiting CRPC cell growth within the context of competing androgen levels. First, we demonstrate that ARCC-4 continues to degrade the AR in VCaP cells in the presence of increasing concentrations of the synthetic androgen R1881 up to 1 nM (Fig. 6a), but ultimately is out-competed for AR binding at higher R1881 concentrations. When VCaP cells were treated with a fixed concentration of ARCC-4, enzalutamide or the epimer in the presence of increasing concentrations of R1881, ARCC-4 outperformed enzalutamide in retaining its antiproliferative and pro-apoptotic potency at higher R1881 concentrations. For instance, at R1881 concentrations above 0.1 nM, 1 µM enzalutamide starts to lose its ability to induce apoptosis or inhibit cell proliferation (Fig. 6b, c). On the other hand, 1 µM ARCC-4 retains its functional activity up to 1 nM R1881. The observed loss of ARCC-4 functional activity at higher R1881 concentrations clearly shows that our previously observed suppression of prostate cancer cell growth by ARCC-4 is indeed mediated through AR signaling. These data demonstrate the superior efficacy of protein degradation over inhibition in conditions that mimic the high androgen environments observed in CRPC tumors. Furthermore, they illustrate that unlike inhibitor-based approaches, the PROTAC technology is sufficient to achieve maximal biological activity despite any anticipated challenges associated with the larger size.

## Discussion

In an attempt to study the potential functional merits of protein degradation over inhibition as a therapeutic strategy, we performed the first head-to-head comparison, to our knowledge, of a drug inhibitor (enzalutamide) and its PROTAC degrader (ARCC-4) using cellular models of drug resistance. Here we demonstrate that AR PROTACs can degrade AR at low nanomolar concentrations in a manner that is independent of the cell line tested. Moreover, we demonstrate that AR PROTACs are particularly suitable in abrogating AR signaling. We show that PROTAC-mediated AR degradation can address the limitations of current AR antagonists used in CRPC therapy. Some of the escape mechanisms associated with resistance to current AR antagonists include AR amplifications, AR mutations, and intra-tumoral androgen synthesis that can out-compete AR antagonists and provide a local source of androgens to propel cell proliferation.

The tool compound ARCC-4 reduces AR protein levels by greater than 95%, even in cells expressing high levels of AR protein. Furthermore, since AR degradation leads to loss of protein function, ARCC-4 is less susceptible to the agonist activity observed upon mutation of its AR binding moiety (e.g., AR-F876L). Our results also show that AR PROTACs can be employed across a wide spectrum of mutations that have emerged due to previous therapies.

Another benefit of PROTACs stems from their remarkable potency, which is due to three factors: a catalytic mode of action, ability to induce favorable protein–protein interactions between the target protein and E3 ligase, and the need to form the active trimer for only brief periods of time^9,37,38^. We believe these three factors are at play in the improved biological potency of ARCC-4 over that of enzalutamide. ARCC-4 is approximately 10-fold more potent than enzalutamide in functional activity assays. Despite sharing the AR binding moiety, the epimer of ARCC-4 is much less potent than enzalutamide, likely reflecting its lower cell permeability, and suggesting that the 10-fold higher potency of ARCC-4 over enzalutamide occurs despite lower intracellular concentrations of ARCC-4. Thus, future PROTACs with enhanced permeability could widen the potency differential with enzalutamide even further.

Despite high plasma concentrations of enzalutamide in mouse models and in a clinical setting, enzalutamide is more efficacious in a castration setting^39–41^ suggesting that physiological (i.e., non-castrated) androgen levels block enzalutamide from inhibiting AR. Considering that both enzalutamide and ARCC-4 bind to the AR ligand binding domain in competition with high-affinity androgens, our data suggest that a PROTAC approach is better able to ablate AR signaling in the presence of androgens.

ARCC-4 will be useful as a tool compound to probe AR biology and dissect the *in vitro* cellular mechanisms of diseases that rely on AR, such as prostate cancer, breast cancer and spinal bulbar muscular atrophy (Kennedy’s disease)^1,42,43^.

Future work to generate compounds with optimal pharmacodynamic and pharmacokinetic properties will allow us to investigate whether the AR PROTAC activity observed in these studies is reflected in in vivo models. In summary, we have designed a potent enzalutamide-based AR PROTAC that allows for a direct comparison between protein degradation and inhibition in CRPC cell lines and moreover demonstrated the advantages of this PROTAC over its parent inhibitor in a number of cell-based models of enzalutamide resistance.

## Methods

### Cell lines, antibodies, and reagents

VCaP, LNCaP, 22Rv1, PC3 and T47D cells were purchased from ATCC. LNCaP/AR and LNCaP/F876L cells were a kind gift from Donald McDonnell at Duke University, Durham. Stable LNCaP cell lines expressing wt-AR (LNCaP-AR) and F876L mutation (LNCaP-F876L), were generated using pQC-XIP retrovirus vector (Promega). AR-expressing HEK293 cells were generated by cloning AR into pDNA/4TO vector (Invitrogen). The plasmids were transfected into HEK293 cells and a pool of stable clones was selected for each construct. Cell lines purchased from ATCC are thoroughly authenticated using short tandem repeat DNA profiling. All cell lines tested negative for mycoplasma contamination. VCaP and AR-expressing 293 T cells were cultured in DMEM medium supplemented with 10% fetal bovine serum (FBS) and 1% penicillin-streptomycin. LNCaP, LNCaP/AR, LNCaP/F876L and T47D cells were cultured in EMEM medium supplemented with 10% FBS and 1% penicillin-streptomycin. PC3 cells were cultured in F-12K medium supplemented with 10% FBS and 1% penicillin-streptomycin. AR (5153), GAPDH (2118), GR (12041), ER (8644) and PR-A/B (8757) antibodies were purchased from Cell Signaling Technology. Tubulin antibody (T9026) was purchased from Sigma and PSA antibody (A0562) was purchased from Agilent Technologies. GAPDH and tubulin antibodies were diluted 1:3000, AR antibody was diluted 1:2000, GR, ER and PR-A/B antibodies were diluted 1:1000 and PSA antibody was diluted 1:500. Epoxomicin synthesis has been previously described^44^. R1881 (R0908) and bafilomycin (19-148) were purchased from Sigma. Enzalutamide (S1250) was purchased from SelleckChem. Charcoal-stripped FBS (FB-04) was purchased from Omega scientific and FBS (04-001-1A-US) was purchased from Biological Industries.

### Western blotting

Cells were lysed in RIPA buffer (50 mM Tris (pH 7.5), 150 mM NaCl, 1% NP-40, 0.5% sodium deoxycholate, 0.1% SDS) supplemented with protease (11697498001, Roche) and phosphatase inhibitors (10 mM sodium fluoride, 10 mM β-glycerophosphate, 2 mM sodium orthovanadate and 10 mM sodium pyrophosphate). Lysates were centrifuged at 15,000 rpm and the supernatants were analyzed by SDS/PAGE. Western blotting was performed by transferring samples onto a nitrocellulose membrane, incubating in 5% milk in TBST (Tris-buffered Saline with Tween 20) at room temperature for 1 h and probing with indicated antibody overnight at 4 °C. Membranes were visualized using the ECL prime western blotting detection reagent (GE Healthcare, RPN2232).

### RNA interference

VCaP cells were transfected with endo-ribonuclease prepared siRNAs against VHL (Sigma, EHU074571) or Firefly Luciferase (Sigma, EHUFLUC) as a negative control. Cells were seeded to 60–80% confluency, then forward transfected using Lipofectamine^TM^ RNAiMAX transfection reagent (ThermoFisher, 13778150). Subsequent experiments were performed 48 h post transfection.

### Tandem Ubiquitin Binding Elements (TUBE1) pull-down assay

VCAP cells were treated with 1 µM of enzalutamide, ARCC-4, epimer or vehicle for 2.5 h at 37 °C. Cells were then lysed in 50 mM Tris-HCl (pH 7.5), 150 mM NaCl, 1 mM EDTA, 1% NP-40, 10% glycerol, supplemented with protease (11697498001, Roche), phosphatase, (10 mM sodium fluoride, 2 mM sodium orthovanadate, and 10 mM β-glycerophosphate) and deubiquitinase inhibitors (10 mM N-ethylmaleimide, 20 µM PR-619) for 10 minutes. Lysates were cleared by centrifugation at 15,000 RPM for 10 minutes. Equal amounts of supernatants were then incubated with 25 µL TUBE1 agarose beads at 4 °C for 2 h. Beads were then collected by centrifugation (5000 RPM for 1 minute), washed and re-suspended in 2 × SDS buffer. Beads were then boiled for 5 minutes and analyzed by SDS/PAGE. Western blotting was performed according to standard protocols.

### Human androgen receptor radioligand binding assay

Agonist radioligand receptor binding assays were performed using LNCaP cell lysates. Experiments were performed by Eurofins Pharma Discovery Services in France according to the previously published AR receptor assay protocol^45^.

### Cell proliferation assays

Cells were seeded in 96-well plates at 5000 cells per well in 150 µL of media and incubated at 37 °C for 3 days. VCaP cells were seeded in phenol red free DMEM + 10% charcoal-stripped FBS (Omega), PC3 cells were seeded in F-12K + 10% FBS and LNCaP, LNCaP/AR, LNCaP/F876L cells were seeded in phenol red free RPMI + 5% charcoal-stripped FBS. Cells were treated with 50 µL of 4 × concentrated compound to yield indicated concentrations for each experiment. Treated cells were incubated at 37 °C for 6 days (PC3 cells were incubated for 4 days) after which CellTiter-Glo reagent (Promega, G9242) was added to plates. Plates were shaken for 2 minutes to lyse cells and incubated at room temperature for 10 minutes. Plates were read on a luminometer and data was analyzed and plotted using GraphPad Prism software.

### Apoptosis assays

VCaP cells were seeded in 96-well plates in phenol red free DMEM + 10% charcoal-stripped FBS at 5000 cells per well in 150 µL of media and incubated at 37 ^o^C for 2 days. Cells were treated with 50 µL of 4 × concentrated compound to yield indicated concentrations for each experiment. Treated cells were incubated at 37 °C for 48 h after which Caspase-Glo 3/7 (Promega, G8091) was added to plates and incubated at room temperature for 1 h. Plates were read on a luminometer and data was analyzed and plotted using GraphPad Prism software.

### Chemical synthesis

Detailed procedures of AR PROTACs syntheses can be found in Supplementary Methods. See Supplementary Figure 1 for synthetic scheme.

### Data availability

All data supporting the findings of this study are available within the paper and Supplementary information files. ^1^H NMR spectra of the compounds described in this study (2a, 2b, 2c, and 3) are presented in Supplementary Figures 12–15. Please address compound requests to T.K.N.: taavi.neklesa@arvinas.com.

## Electronic supplementary material

Supplementary Information
