# Supplementary material for: Androgen receptor degradation by the proteolysis-targeting chimera ARCC-4 outperforms enzalutamide in cellular models of prostate cancer drug resistance
Source: Commun Biol. 2018 Aug 2;1:100. doi: 10.1038/s42003-018-0105-8 (PMC6123676; doi:10.1038/s42003-018-0105-8)
Supplement: Supplementary file 1 — Supplementary Information [file 42003_2018_105_MOESM1_ESM.pdf]

Supplementary Information

Supplementary Figures

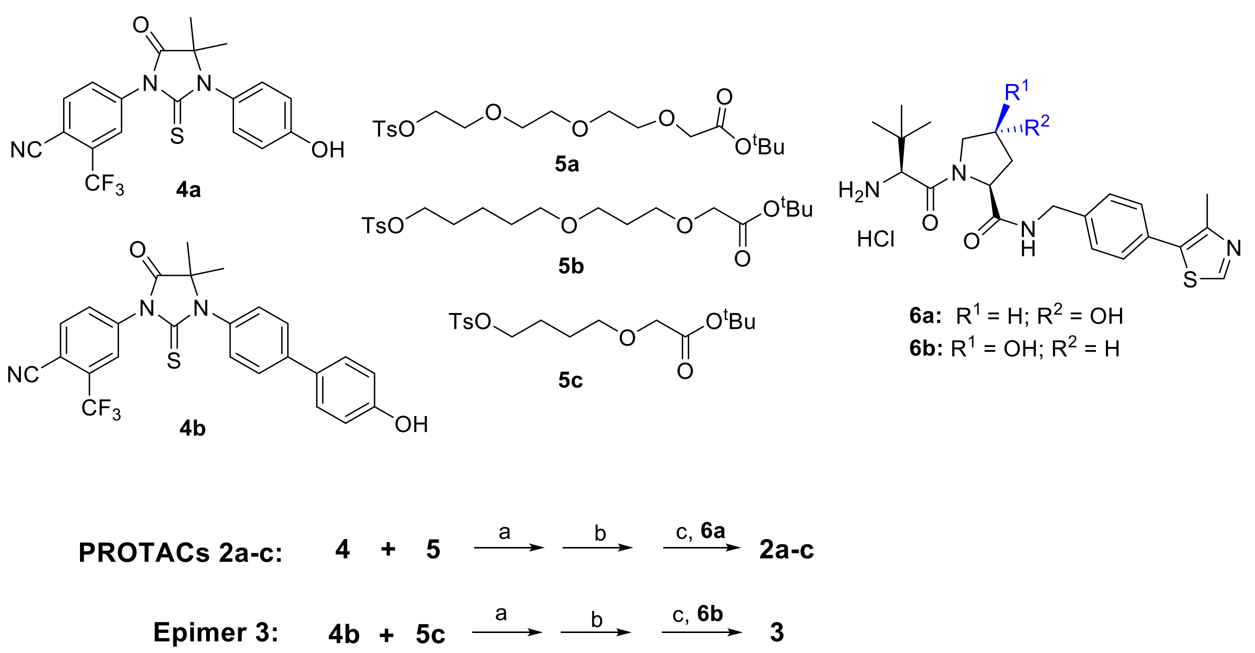

Supplementary Figure 1. Synthesis of AR PROTACs. Compounds **2a-c** and **3** were assembled from three components. AR intermediates **4a** and **4b** were prepared as described by Jung, *et al.* (2010) [1], while the preparation of linker intermediates **5a-c** and VHL intermediates **6a-b** was described in Crew, *et al.* (2018) [2] Reagents and conditions: a) K_2CO_3, DMF, 80 °C; b) 2N HCl/dioxane; c) EDCI, HOBt, DMF, rt.

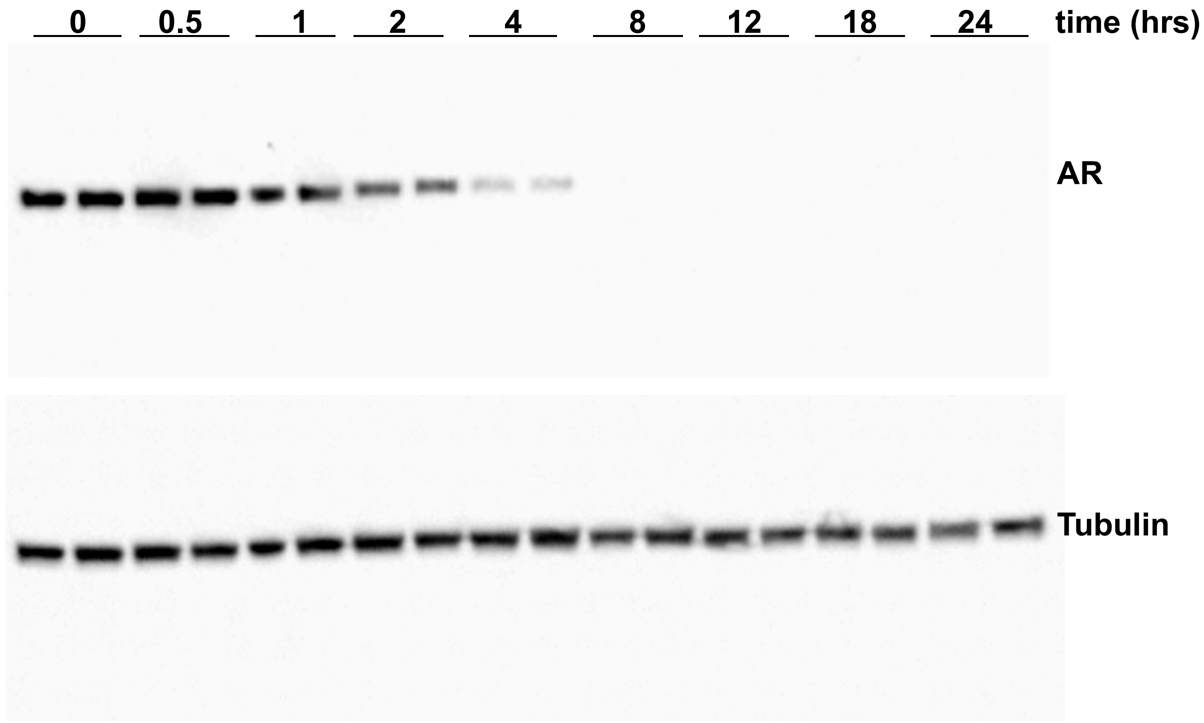

Supplementary Figure 2. Time course of ARCC-4-induced AR degradation in VCaP cells. Western blot image for time course experiment with serum-free treatment of VCaP cells with 100nM of ARCC-4. Image shows biological replicates and is representative of two independent experiments (n=2).

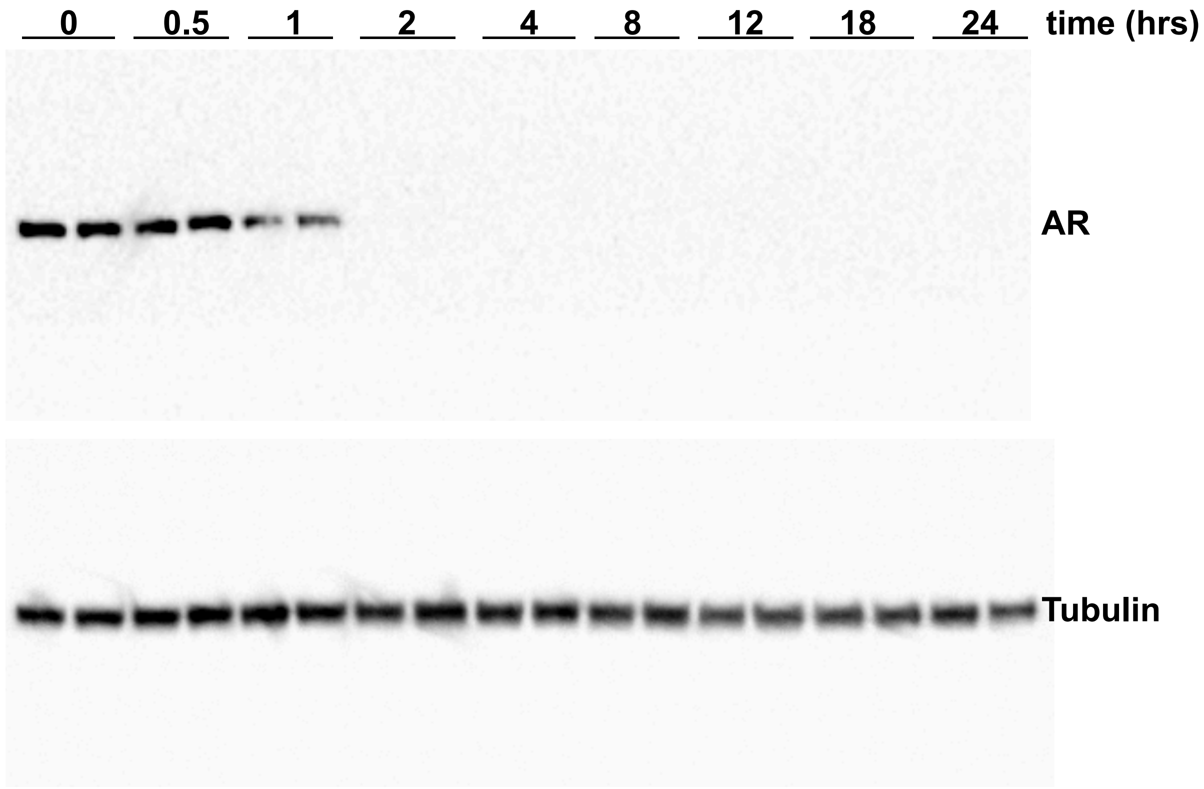

Supplementary Figure 3. Time course of ARCC-4-induced AR degradation in LNCaP cells. Western blot image for time course experiment treating LNCaP cells with 100nM of ARCC-4 in 5% charcoal stripped serum media. Image shows biological replicates and is representative of two independent experiments (n=2).

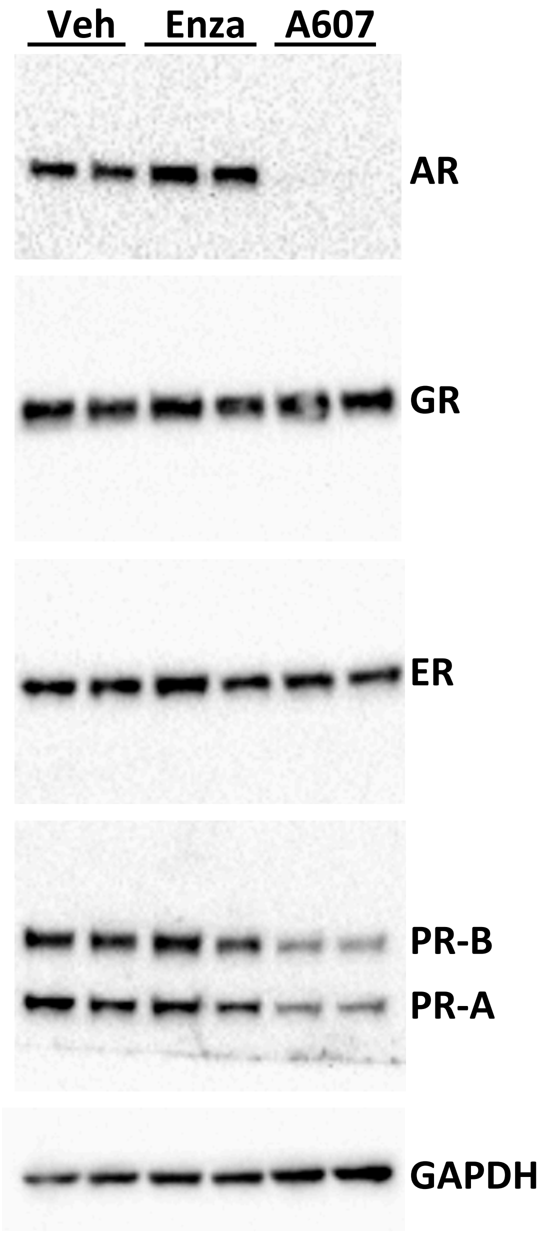

Supplementary Figure 4. Selectivity of ARCC-4 for different nuclear hormone receptors. Western blot image for 24-hour treatment of T47D breast cancer cells with 1 μM enzalutamide (Enza), ARCC-4 or vehicle (Veh). Slight decrease in progesterone receptors (PR-A and PR-B) levels is observed but glucocorticoid receptor (GR) and estrogen receptor (ER) levels are unaffected. Image shows biological replicates and is representative of two independent experiments.

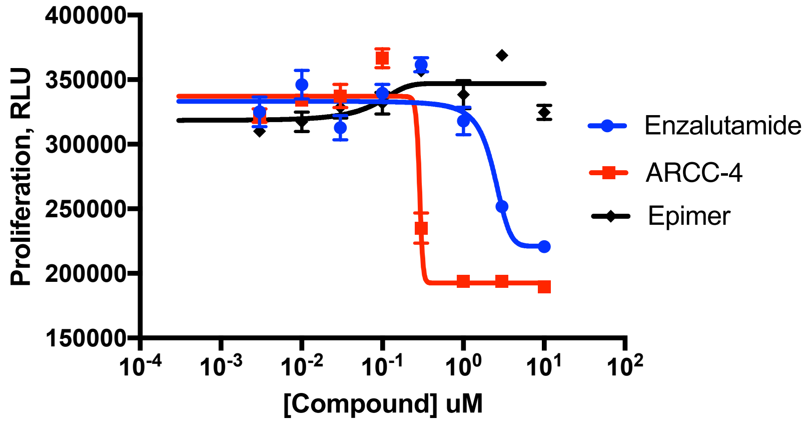

Supplementary Figure 5. Inhibition of proliferation in LNCaP cells. Treatment of LNCaP cells with enzalutamide, ARCC-4, or epimer for 6 days has antiproliferative effects. Experiments were performed in triplicate.

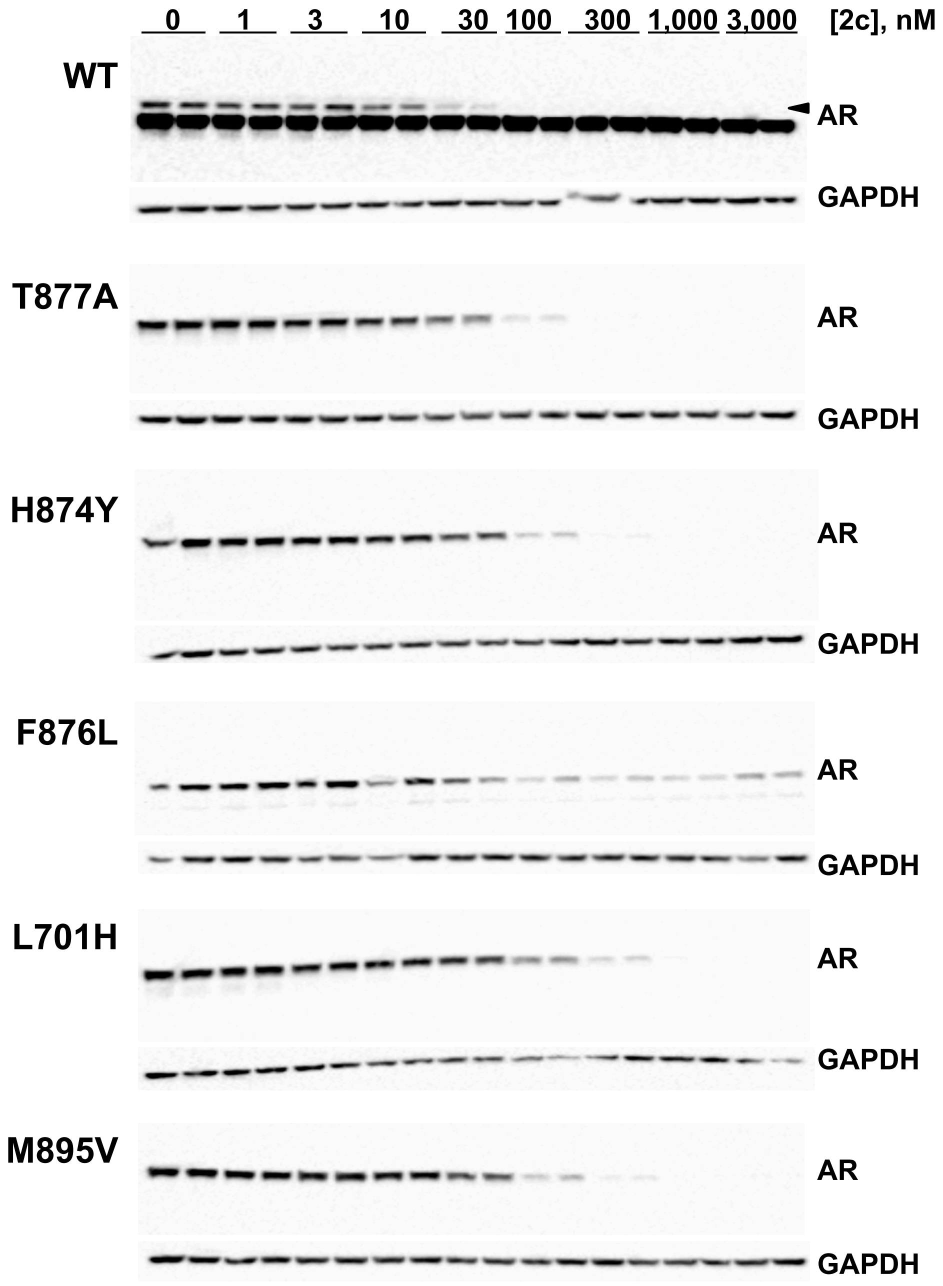

Supplementary Figure 6. Dose response of ARCC-4-induced AR degradation in drug resistant AR mutants. Western blot image for treatment of HEK293T cells overexpressing wild type (WT) or different AR mutants. AR expression was induced with an 18hr treatment with 50ng per ml doxycycline induction after which cells were treated with ARCC-4 for 20hrs in 5% charcoal stripped serum media. Experiments were performed in duplicate and the plot is representative of two independent experiments (n=2).

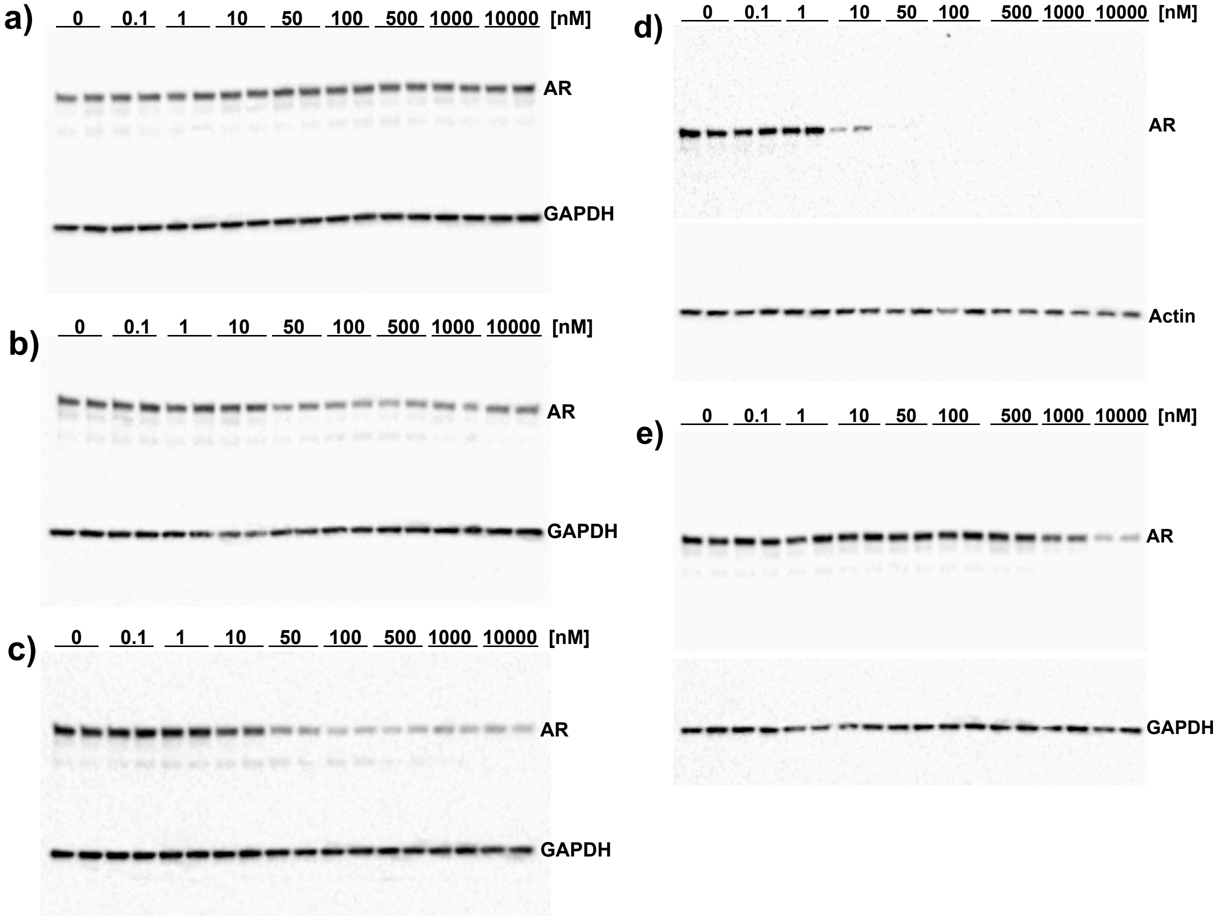

Supplementary Figure 7. Full blot images showing AR degradation for different compounds. Treatment of VCaP cells with increasing concentrations of (a) enzalutamide (b) compound **2a** (c) compound **2b** (d) compound **2c** and, (e) SARD279.

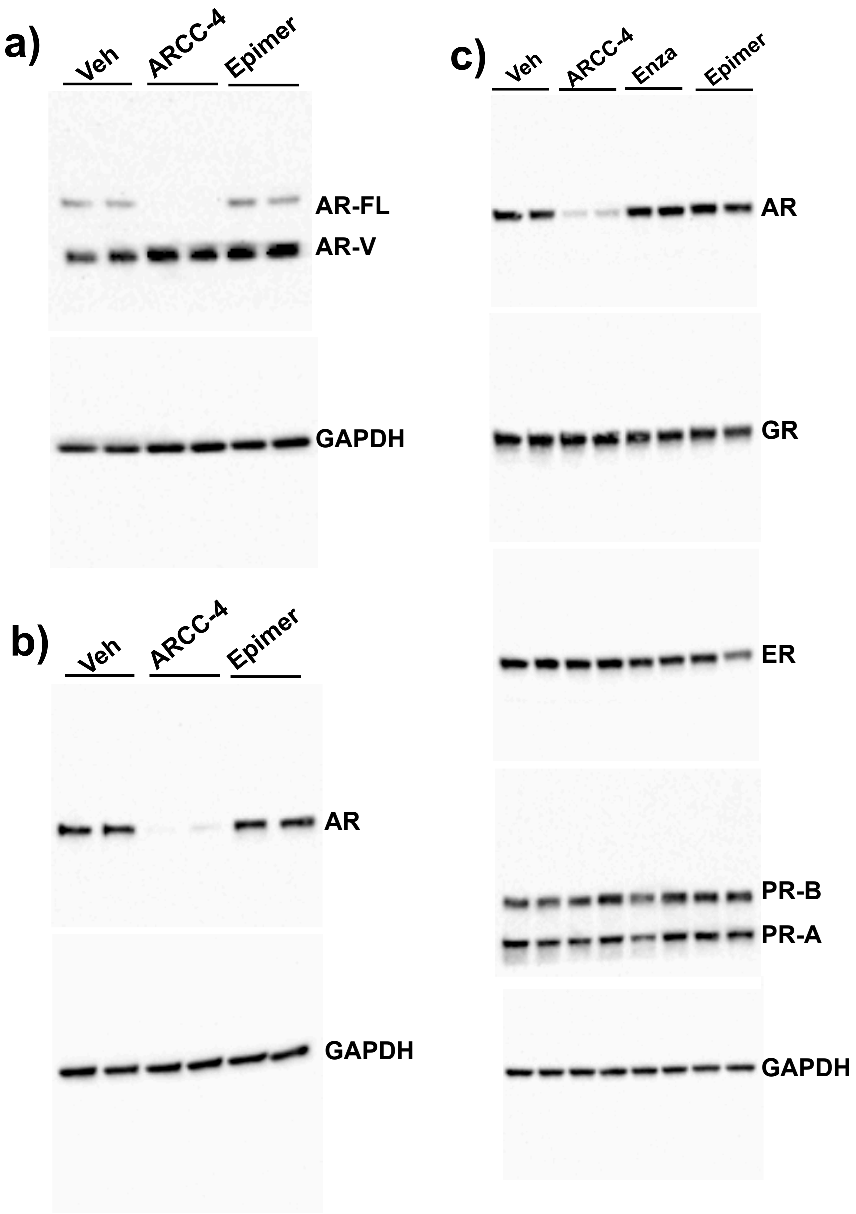

Supplementary Figure 8. Full blot images of ARCC-4 activity in different cell lines. (a) Treatment of 22RV1 cells with 100 nM of vehicle (veh), ARCC-4 or epimer. (b) Treatment of LNCaP cells with 100 nM of vehicle (veh), ARCC-4 or epimer. (c) Treatment of T47D cells with 50 nM of vehicle (veh), ARCC-4, enzalutamide (enza) or epimer.

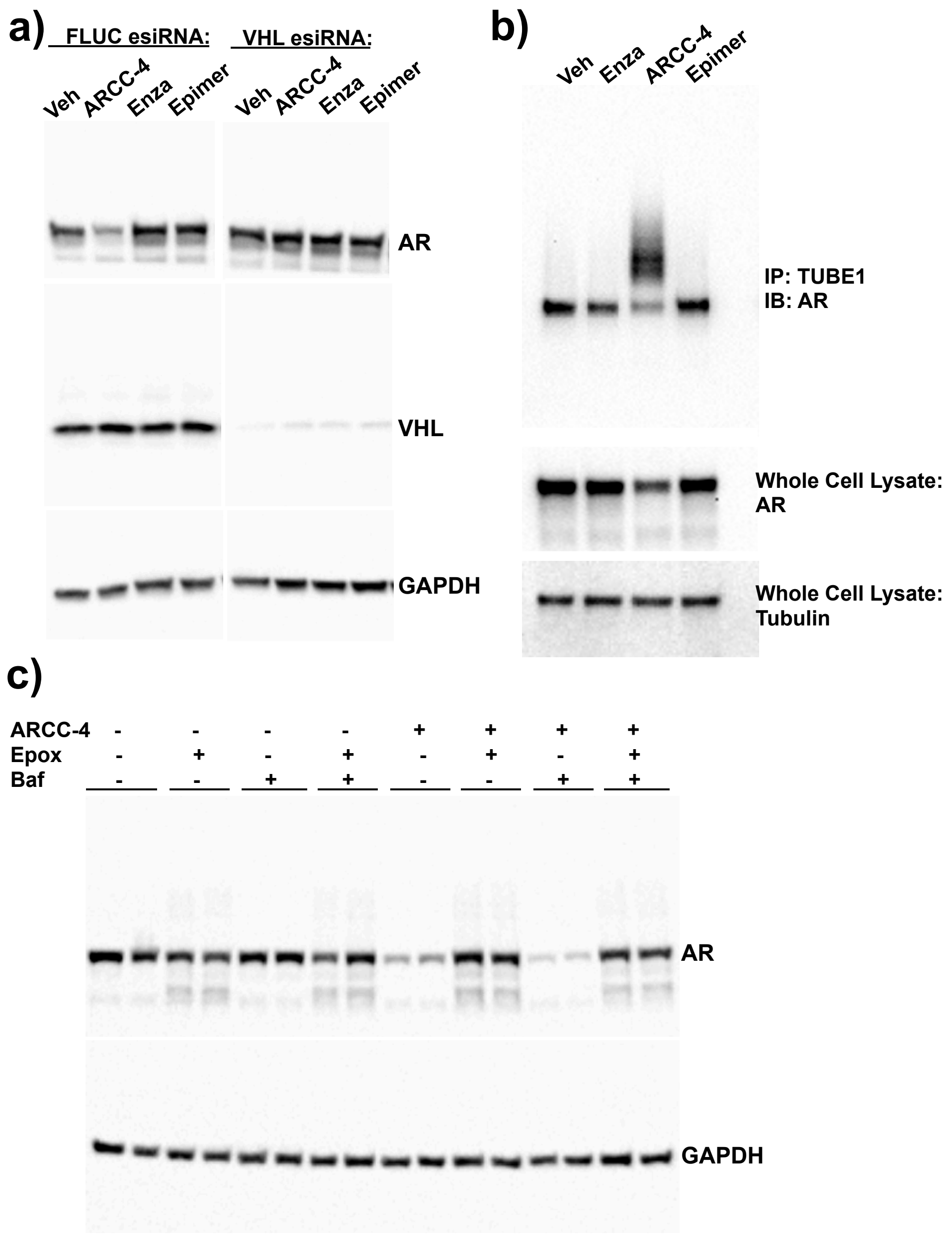

Supplementary Figure 9. Full blot images showing ARCC-4 mechanism of action in VCaP cells. (a) VHL knockdown in VCaP cells. (b) AR polyubiquitination assay. (c) Treatment of VCaP cells with proteasome inhibitor epoxomicin (epox) or lysosomal inhibitor bafilomycin (baf).

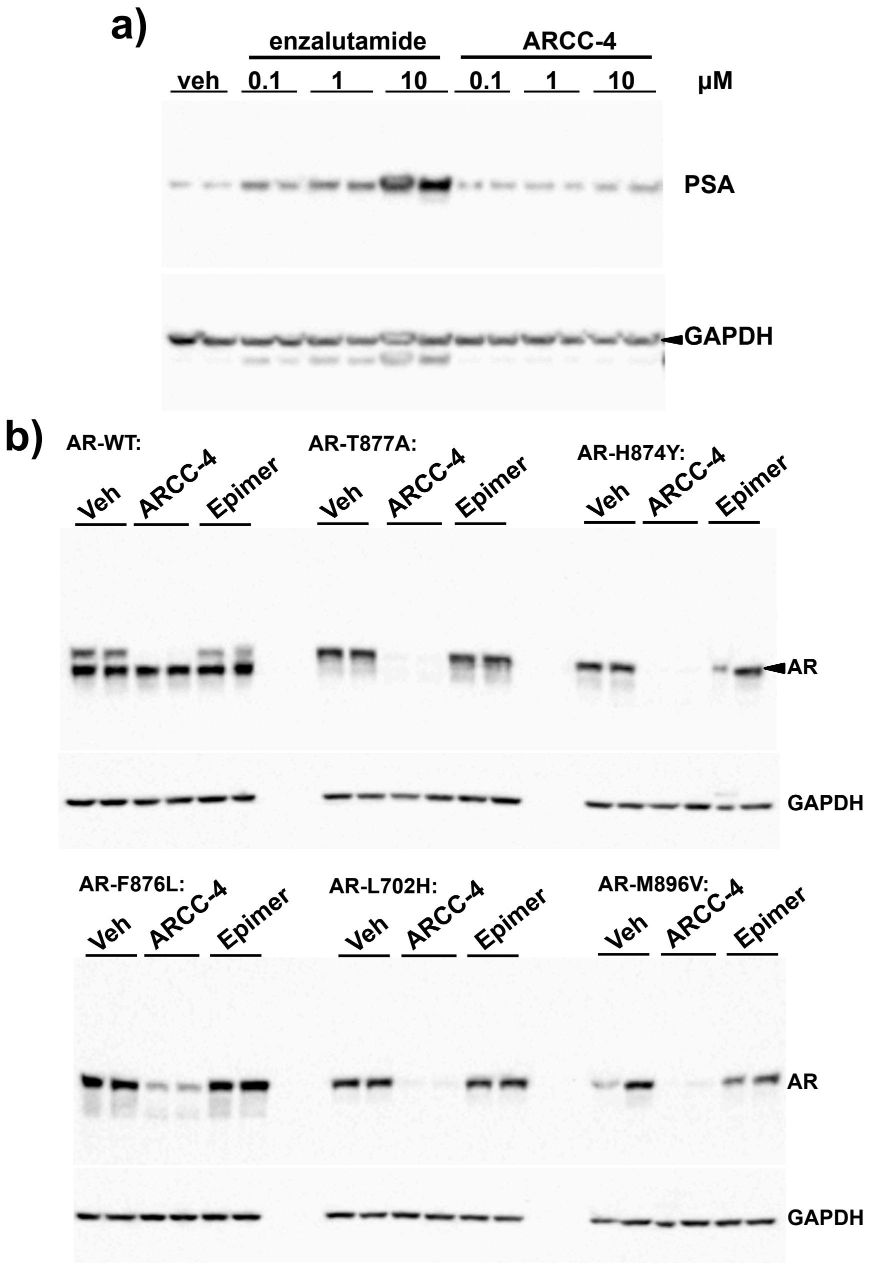

Supplementary Figure 10. Full blot images of ARCC-4 activity against AR mutations. (a) Treatment of LNCaP/F876L cells. (b) Treatment of HEK293T cells overexpressing different AR mutants.

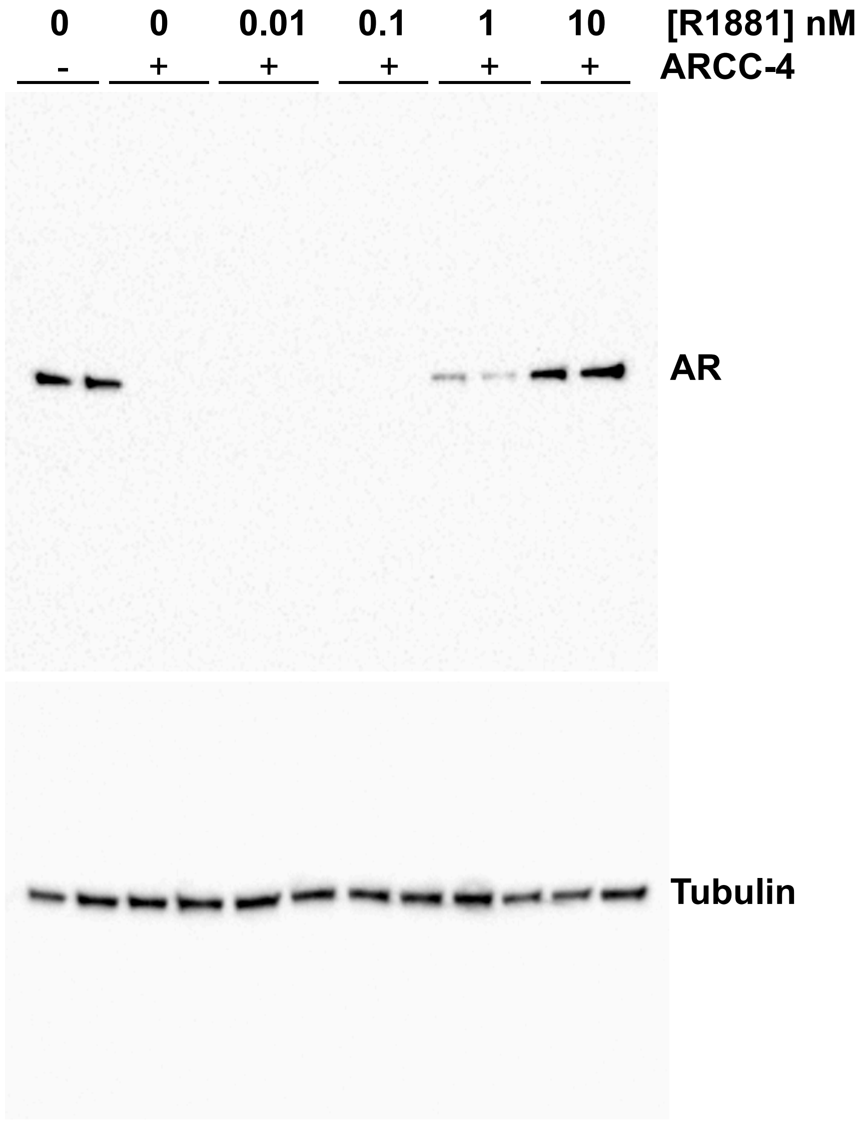

Supplementary Figure 11. Full blot images for AR degradation in the presence of synthetic androgen R1881.

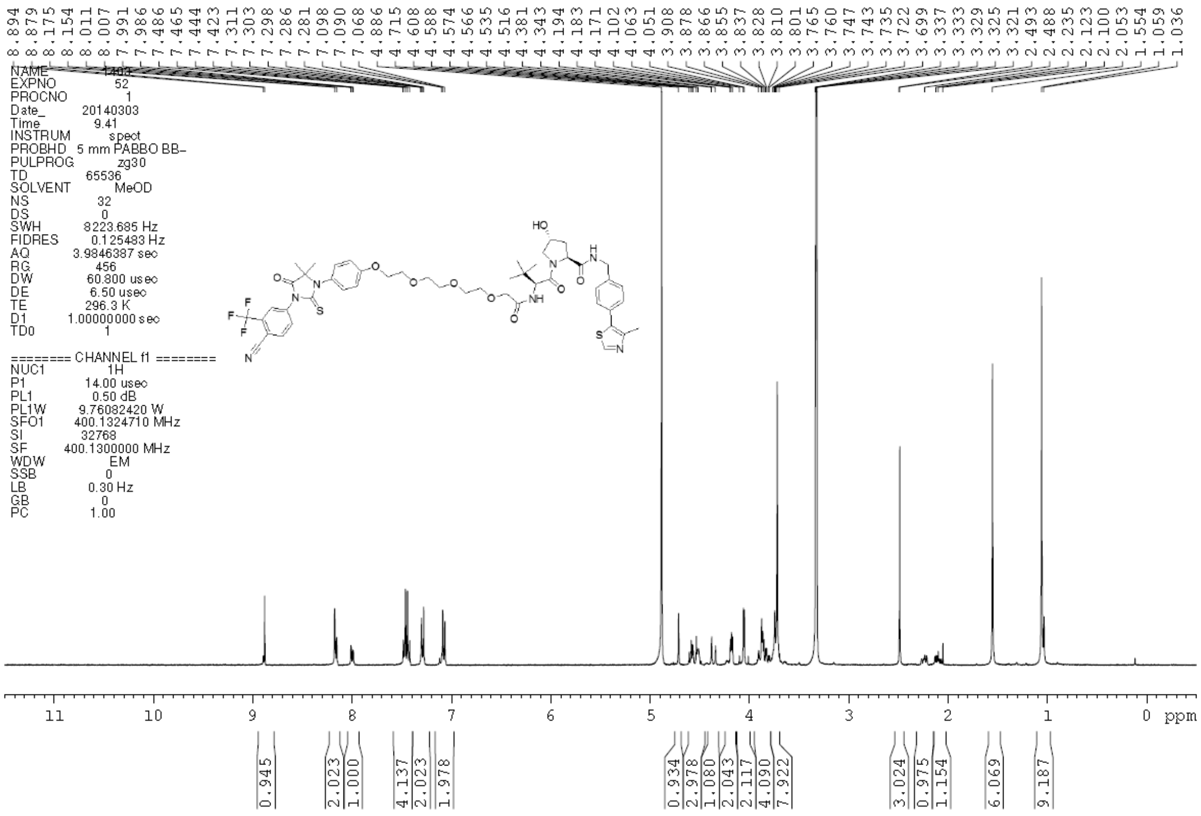

Supplementary Figure 12. ^{1}H NMR spectrum of compound **2a**.

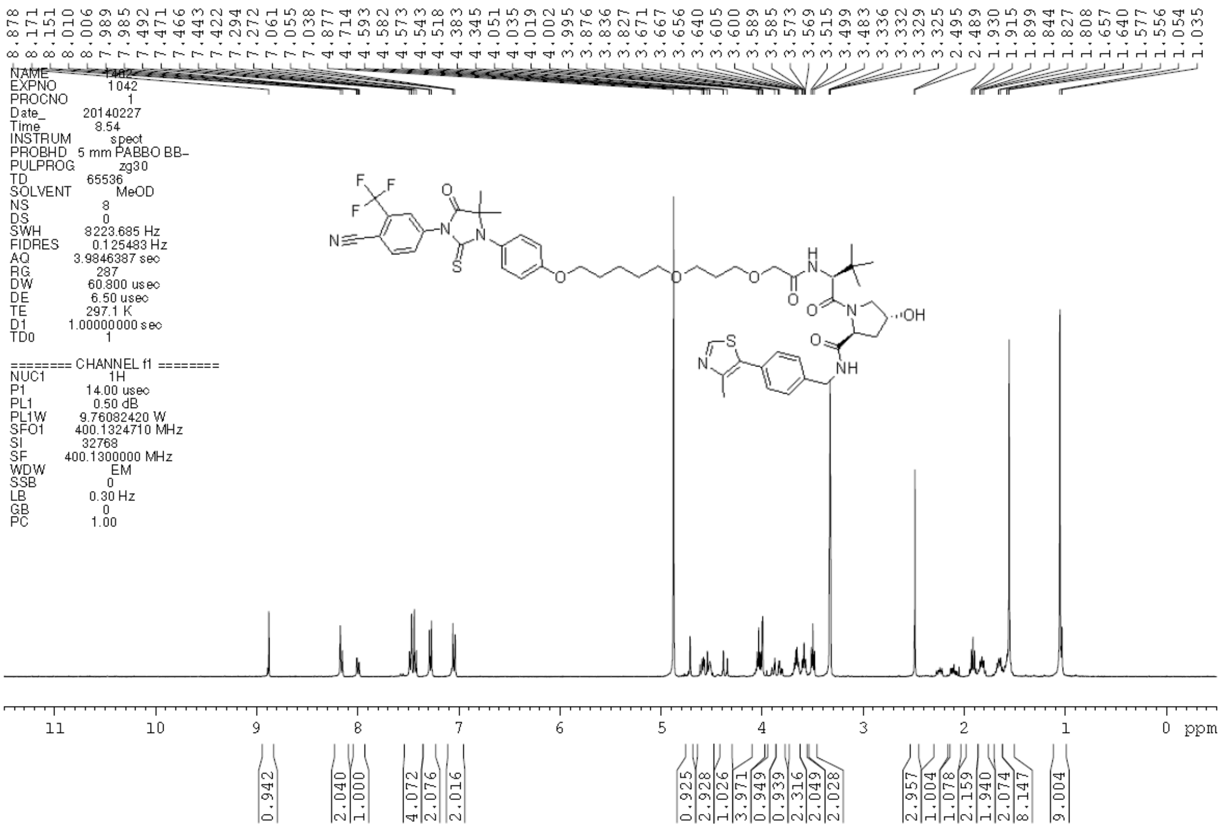

Supplementary Figure 13. ^{1}H NMR spectrum of compound 2b.

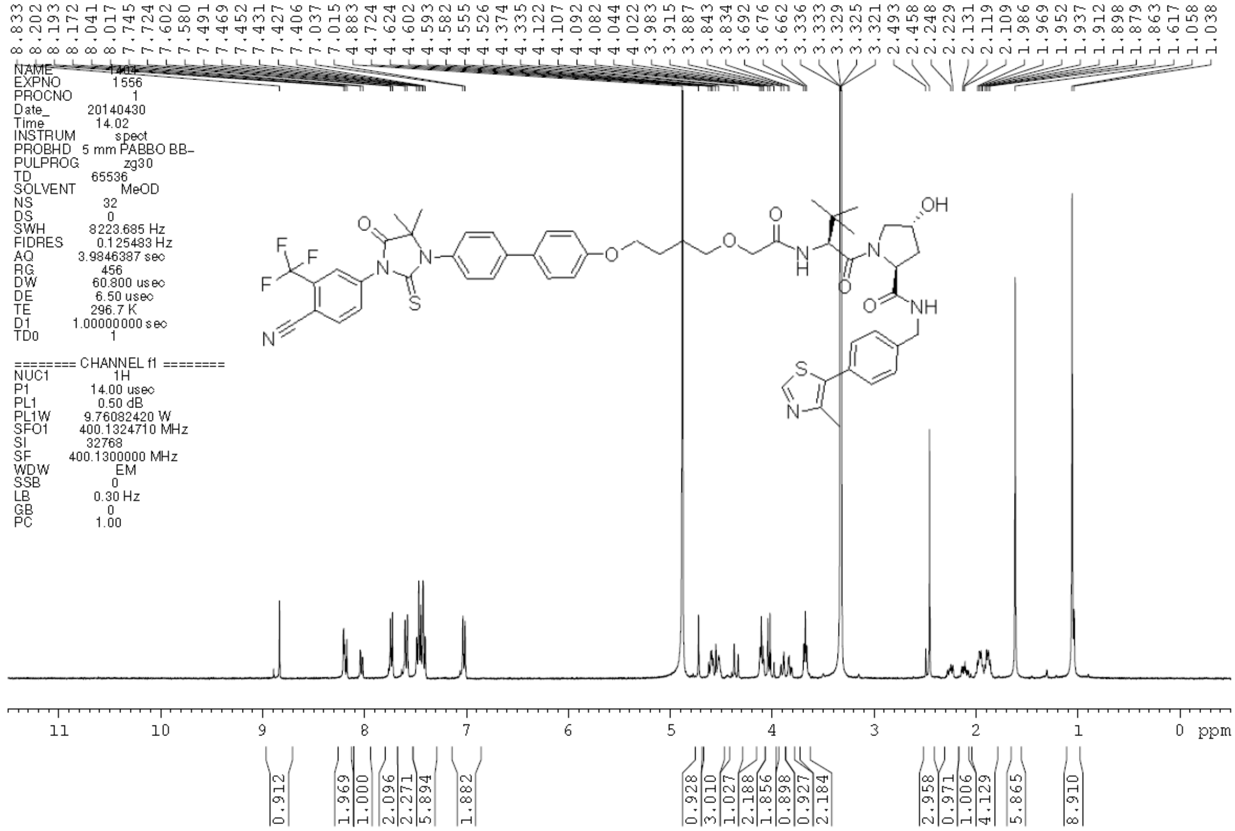

Supplementary Figure 14. ^{1}H NMR spectrum of compound 2c.

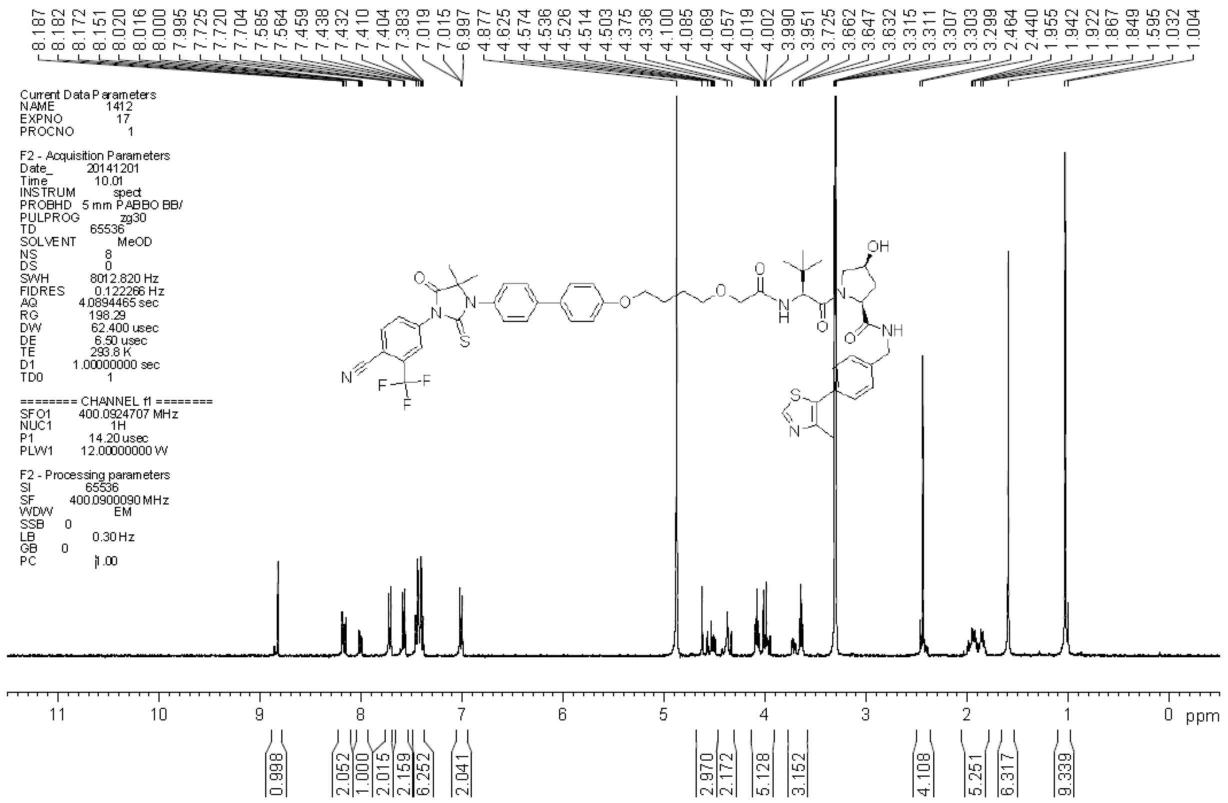

Supplementary Figure 15. ^{1}H NMR spectrum of compound 3.

Supplementary Tables

**Compound**	**IC_{50} (nM)**
R1881	1.6
Enzalutamide	70
ARCC-4	36
Epimer	41

Supplementary Table 1. Human AR (agonist radioligand) Receptor Binding Assay. Table of IC50 values from AR radioligand binding assays. The IC50 values represent the concentration of each compound that causes half-maximal inhibition of binding between AR and radiolabelled R1881. Binding assays were performed by Eurofins Pharma Discovery Services, France.

Supplementary Methods.

4-[3-(4-hydroxyphenyl)-4,4-dimethyl-5-oxo-2-sulfanylideneimidazolidin-1-yl]-2-(trifluoromethyl)benzonitrile (**4a**): ^{1}H NMR (300 MHz, CD_3OD): δ 8.15 (s, 1H), 8.13 (d, *J* = 8.1 Hz, 1H), 7.97 (dd, *J* = 8.1, 1.5 Hz, 1H), 7.20-7.15 (m, 2 H), 6.94-6.88 (m, 2 H), 1.54 (s, 6 H); LC-MS (ES^{+}): *m/z* 406.0 [MH^{+}].

4-[3-[4-(4-hydroxyphenyl)phenyl]-4,4-dimethyl-5-oxo-2-sulfanylideneimidazolidin-1-yl]-2-(trifluoromethyl)benzonitrile (**4b**): ^{1}H NMR (400 MHz, CD_3OD): δ 8.19 (d, *J* = 1.6 Hz, 1H), δ 8.16 (d, *J* = 8.0 Hz, 1H), 8.02 (dd, *J* = 8.0, 1.6 Hz, 1H), 7.73 (d, *J* = 8.8 Hz, 2H), 7.54 (m, 2H), 7.41 (d, *J* = 8.4 Hz, 2H), 6.91 (m, 2H), 1.60 (s, 6H); LC-MS (ES^{+}): *m/z* 482.0 [MH^{+}].

(2S,4R)-1-[(2S)-2-[2-(3-[[5-(4-[3-[4-cyano-3-(trifluoromethyl)phenyl]-5,5-dimethyl-4-oxo-2-sulfanylideneimidazolidin-1-yl]phenoxy)pentyl]oxy]propoxy)acetamido]-3,3-dimethylbutanoyl]-4-hydroxy-N-[[4-(4-methyl-1,3-thiazol-5-yl)phenyl]methyl]pyrrolidine-2-carboxamide (**2b**)

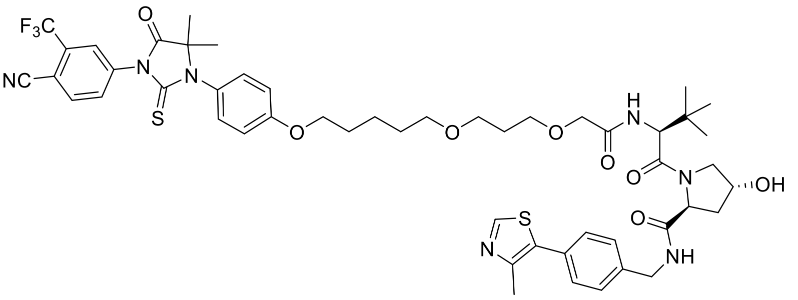

A mixture of 4-[3-(4-hydroxyphenyl)-4,4-dimethyl-5-oxo-2-sulfanylideneimidazolidin-1-yl]-2-(trifluoromethyl) benzonitrile **4a** (141 mg, 0.35 mmol), *tert*-butyl 2-[3-[(5-[[(4-methylbenzene)sulfonyl]oxy]pentyl)oxy]propoxy] acetate **5b** (150 mg, 0.35 mmol), and potassium carbonate (144 mg, 1.04 mmol) in acetonitrile (10 mL) was stirred at 80 °C overnight. The reaction mixture was quenched with water (5 mL), then extracted with ethyl acetate (20 mL

x 2). The combined organic layers were washed with brine, dried over anhydrous sodium sulfate, and concentrated under reduced pressure. The residue was purified by silica gel chromatography with ethyl acetate/petroleum ether (v/v = 1:1), affording the desired product *tert*-butyl 2-(3-[[5-(4-[3-[4-cyano-3-(trifluoromethyl)phenyl]-5,5-dimethyl-4-oxo-2-sulfanylideneimidazolidin-1-yl]phenoxy)pentyl]oxy]propoxy)acetate as yellow oil, 220 mg, 95% yield. LC-MS (ES^+): *m/z* 686.3 [MNa^+].

A solution of *tert*-butyl 2-(3-[[5-(4-[3-[4-cyano-3-(trifluoromethyl)phenyl]-5,5-dimethyl-4-oxo-2-sulfanylideneimidazolidin-1-yl]phenoxy)pentyl]oxy]propoxy)acetate (220 mg, 0.33 mmol) in 2N HCl/dioxane (4 mL) was stirred at 80 °C for 2 h. The resulting mixture was concentrated under reduced pressure to afford 2-(3-[[5-(4-[3-[4-cyano-3-(trifluoromethyl)phenyl]-5,5-dimethyl-4-oxo-2-sulfanylideneimidazolidin-1-yl]phenoxy)pentyl]oxy]propoxy)acetic acid as light-yellow oil. 200 mg, 99% yield. LC-MS (ES^+): *m/z* 608.2 [MH^+].

To a solution of 2-(3-[[5-(4-[3-[4-cyano-3-(trifluoromethyl)phenyl]-5,5-dimethyl-4-oxo-2-sulfanylideneimidazolidin-1-yl]phenoxy)pentyl]oxy]propoxy)acetic acid (160 mg, 0.26 mmol) in N,N-dimethylformamide (5 mL) were added N-(3-dimethylaminopropyl)-N'-ethylcarbodiimide hydrochloride (101 mg, 0.53 mmol), 1-hydroxybenzotrizole (70 mg, 0.52 mmol), N,N-diisopropylethylamine (102 mg, 0.79 mmol). The resulting mixture was stirred at room temperature for 30 mins, then (2S,4R)-1-[(2S)-2-amino-3,3-dimethylbutanoyl]-4-hydroxy-N-[[4-(4-methyl-1,3-thiazol-5-yl)phenyl]methyl]pyrrolidine-2-carboxamide **6a** (170.0 mg, 0.39 mmol) was added. The resulting solution was stirred at room temperature overnight. After the reaction was quenched by the addition of water (5 mL), it was extracted with ethyl acetate (20 mL x 2). The combined organic layers were washed with brine, dried over anhydrous sodium sulfate, and concentrated under reduced pressure. The residue was purified by Prep-HPLC using the

following conditions: Column, XBridge Prep C_{18} OBD Column, 19x150 mm 5 um 13 nm; Mobile phase, water with 10 mM ammonium bicarbonate and acetonitrile (60.0% acetonitrile up to 80.0% in 10 min); Detector, UV 254 nm. The desired product (2S,4R)-1-[(2S)-2-[2-(3-[[5-(4-[3-[4-cyano-3-(trifluoromethyl)phenyl]-5,5-dimethyl-4-oxo-2-sulfanylideneimidazolidin-1-yl]phenoxy)pentyl]oxy]propoxy)acetamido]-3,3-dimethylbutanoyl]-4-hydroxy-N-[[4-(4-methyl-1,3-thiazol-5-yl)phenyl]methyl]pyrrolidine-2-carboxamide **2b** was obtained as a white solid, 60 mg, 22% yield. ^{1}H NMR (400 MHz, CD_3OD): δ 8.88 (s, 1H), 8.17 (s, 1H), 8.16 (d, *J* = 8.0 Hz, 1H), 8.00 (dd, *J* = 8.0, 1.6 Hz, 1H), 7.48, 7.43 (AB, J_{AB} = 8.4 Hz, 4H), 7.28 (d, *J* = 8.8 Hz, 2H), 7.05 (d, *J* = 8.8 Hz, 2H), 4.71 (s, 1H), 4.59-4.52 (m, 3H), 4.37 (d, *J* = 15.2 Hz, 1H), 4.05-4.00 (m, 4H), 3.84 (m, 1H), 3.66 (m, 1H), 3.60 (m, 2H), 3.58 (m, 2H), 3.50 (m, 2H), 2.49 (s, 3H), 2.25 (m, 1H), 2.15 (m, 1H), 1.93-1.81 (m, 4H), 1.66-1.56 (m, 4H), 1.56 (s, 6H), 1.05 (s, 9H); LC-MS (ES^+): *m/z* 1020.3 [MH^+].

Compounds **2a**, **2c** and **3** were similarly prepared.

(2S,4R)-1-[(2S)-2-[1-(4-[3-[4-cyano-3-(trifluoromethyl)phenyl]-5,5-dimethyl-4-oxo-2-sulfanylideneimidazolidin-1-yl]phenyl)-1,4,7,10-tetraoxadodecan-12-amido]-3,3-dimethylbutanoyl]-4-hydroxy-N-[[4-(4-methyl-1,3-thiazol-5-yl)phenyl]methyl]pyrrolidine-2-carboxamide (**2a**)

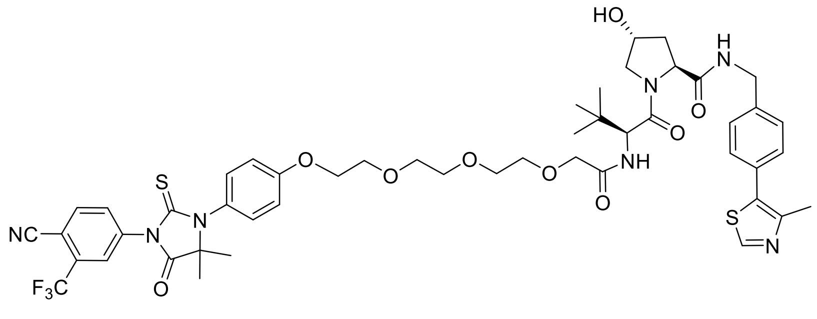

A light-yellow solid. ^{1}H NMR (400 MHz, CD_3OD) δ 8.88 (s, 1H), 8.18 (s, 1H), 8.16 (d, J = 8.4 Hz, 1H), 8.00 (dd, J = 8.0, 1.6 Hz, 1H), 7.48, 7.43 (AB, J_{AB} = 8.4 Hz, 4H), 7.29 (m, 2H), 7.08 (m, 2H), 4.72 (s, 1H), 4.61-4.52 (m, 3H), 4.36 (d, J = 15.2 Hz, 1H), 4.19-4.17 (m, 2H), 4.10-4.05 (m, 2H), 3.91-3.80 (m, 4H), 3.77-3.70 (m, 8H), 2.49 (s, 3H), 2.24-2.05 (m, 2H), 1.55 (s, 6H), 1.06 (s, 9H); LC-MS (ES^+): *m/z* 1008.5 [MH^+].

(2S,4R)-1-[(2S)-2-(2-[4-[4-(4-[3-[4-cyano-3-(trifluoromethyl)phenyl]-5,5-dimethyl-4-oxo-2-sulfanylideneimidazolidin-1-yl]phenyl)phenoxy]butoxy]acetamido)-3,3-dimethylbutanoyl]-4-hydroxy-N-[[4-(4-methyl-1,3-thiazol-5-yl)phenyl]methyl]pyrrolidine-2-carboxamide (**2c**)

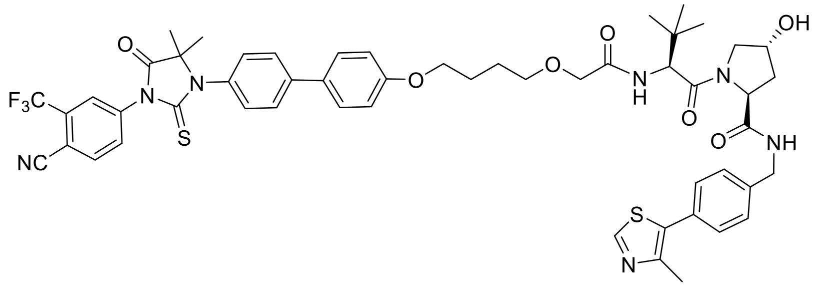

A white solid. ^{1}H NMR (400 MHz, CD_3OD): δ 8.83 (s, 1H), 8.20 (s, 1H), 8.18 (d, J = 8.4 Hz, 1H), 8.03 (d, J = 8.4 Hz, 1H), 7.73 (d, J = 8.4 Hz, 2H), 7.59 (d, J = 8.8 Hz, 2H), 7.49-7.41 (m, 6H), 7.02 (d, J = 8.8 Hz, 2H), 4.72 (s, 1H), 4.62-4.53 (m, 3H), 4.35 (d, J = 15.6 Hz, 1H), 4.12-4.08 (m, 4H), 4.04-3.83 (m, 2H), 3.69-3.66 (m, 2H), 2.46 (s, 3H), 2.24 (m, 1H), 2.12 (m, 1H), 1.99-1.86 (m, 4H), 1.62 (s, 6H), 1.06 (s, 9H); LC-MS (ES^+): m/z 1024.3 [MH^+].

(2S,4S)-1-[(2S)-2-(2-[4-[4-(4-[3-[4-cyano-3-(trifluoromethyl)phenyl]-5,5-dimethyl-4-oxo-2-sulfanylideneimidazolidin-1-yl]phenyl)phenoxy]butoxy]acetamido)-3,3-dimethylbutanoyl]-4-hydroxy-N-[[4-(4-methyl-1,3-thiazol-5-yl)phenyl]methyl]pyrrolidine-2-carboxamide (**3**)

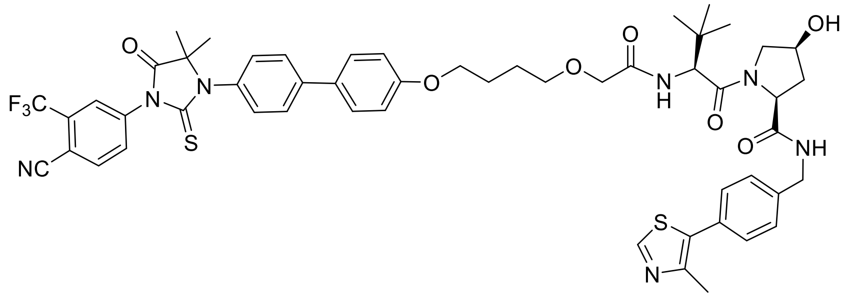

A white solid. ^{1}H NMR (400 MHz, CD_3OD) δ 8.82 (s, 1H), 8.18 (d, *J* = 2.0 Hz, 1H), 8.16 (d, *J* = 8.4 Hz, 1H), 8.01 (dd, *J* = 8.4, 2.0 Hz, 1H), 7.71 (d, *J* = 8.4 Hz, 2H), 7.57 (d, *J* = 8.4 Hz, 2H), 7.46-7.38 (m, 6H), 7.01 (d, *J* = 8.8 Hz, 2H), 4.63 (m, 1H), 4.57-4.50 (m, 2H), 4.38-4.34 (m, 2H), 4.10-3.95 (m, 5H), 3.73-3.63 (m, 3H), 2.46 (s, 3H), 2.45 (m, 1H), 1.96-1.85 (m, 5H), 1.60 (s, 6H), 1.00 (s, 9H); LC-MS (ES^+): *m/z* 1024.2 [MH^+].

Supplementary References

1. **Jung ME, *et al.* Structure−Activity Relationship for Thiohydantoin Androgen Receptor Antagonists for Castration-Resistant Prostate Cancer (CRPC). *Journal of Medicinal Chemistry* 53, 2779-2796 (2010).**

2. **Crew AP, *et al.* Identification and Characterization of Von Hippel-Lindau-Recruiting Proteolysis Targeting Chimeras (PROTACs) of TANK-Binding Kinase 1. *Journal of Medicinal Chemistry* 61, 583-598 (2018).**
